# Spontaneous intracapsular hemorrhage of a giant hepatic cavernous hemangioma: a rare case report and literature review

**DOI:** 10.1186/s12876-021-01666-z

**Published:** 2021-02-23

**Authors:** Yong-Guang Yang, Wei-Feng Chen, Wei-Heng Mai, Xiao-Fang Li, Hong-Lian Zhou, Li-Juan Liu, Ming-Yi Li

**Affiliations:** 1 Department of Hepatobiliary Surgery, The Affiliated Hospital of Guangdong Medical University, Zhanjiang, Guangdong People’s Republic of China; 2 Department of Pathology, The Affiliated Hospital of Guangdong Medical University, Zhanjiang, Guangdong People’s Republic of China; 3 Department of Ultrasound Diagnostics, Guangdong Province, The Affiliated Hospital of Guangdong Medical University, 57th, Renmin South Road, Xia shan District, Zhanjiang, 524001 People’s Republic of China

**Keywords:** Hepatic hemangioma, Intracapsular hemorrhage, Magnetic resonance imaging

## Abstract

**Background:**

Hepatic cavernous hemangioma is the most common type of benign liver tumor. Although ruptures and hemorrhages of hepatic hemangioma are rare complications, they are associated with high mortality. Most practitioners only pay more attention to abdominal hemorrhages caused by the rupture of hepatic hemangiomas. However, spontaneous intracapsular hemorrhages can often be neglected and poorly understood.

**Case presentation:**

A 65-year-old man was referred to our institution with right upper quadrant pain, which had occurred suddenly and without a history of recent trauma. The blood test results were normal. Magnetic resonance imaging (MRI) of the abdomen showed a cystic mass in the right liver lobe. Considering the possibility of hepatic cystadenoma with hemorrhage, the patient underwent a right hepatic lobectomy. The pathological findings unexpectedly revealed intratumoral hemorrhage of hepatic hemangioma. The patient recovered well and was discharged eight days after surgery.

**Conclusions:**

Intracapsular hemorrhage of hepatic cavernous hemangioma is challenging to diagnose and has a high potential risk of rupture. MRI is beneficial for diagnosing subacute internal hemorrhage cases, and it is recommended to undergo surgery for patients with a definitive diagnosis.

## Background

The cavernous liver hemangioma is the most common benign tumor of the liver, and its pathogenesis is still unclear. It is common in middle-aged women [1]. Most of the patients with hepatic hemangioma have no clinical symptoms and are only followed up. Treatment is necessary when the tumor size increases to the point of causing symptoms such as abdominal discomfort, vomiting, poor appetite, even serious complications such as tumor rupture or bleeding [2–4]. Although hepatic hemangioma rupture and hemorrhage are uncommon complications, they can be lethal [5, 6]. Spontaneous intracapsular hemorrhage of cavernous hepatic hemangioma is rare. There are only eight Chinese or English reports in the past 26 years [7–14], which causes a lack of in-depth understanding. The author reports a patient diagnosed pathologically with intracapsular hemorrhage of hepatic hemangioma and reviews the relevant literature at home and abroad, which could help us better understand it.

## Case presentation

**Fig. 1:**
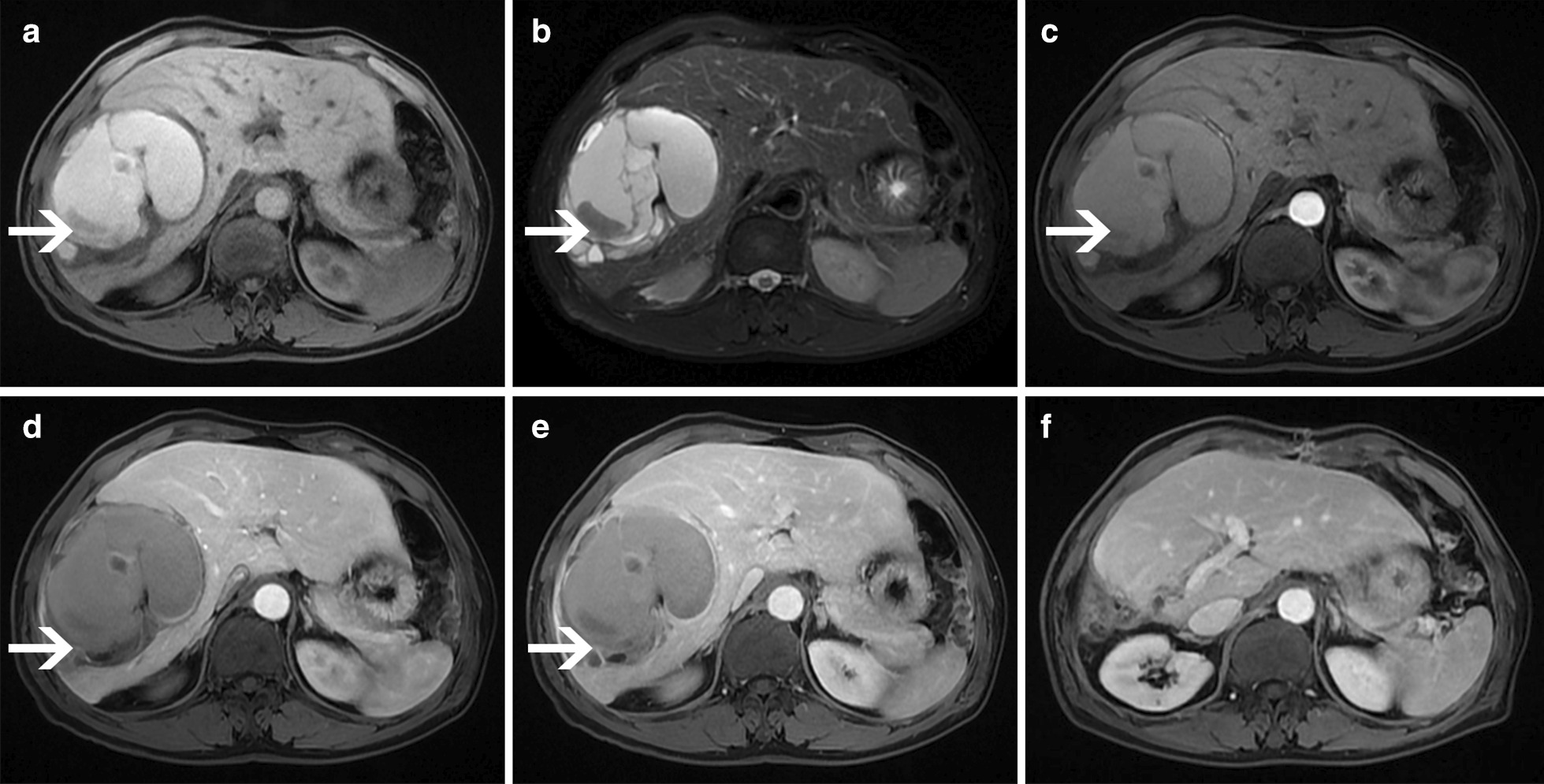
MRI examination. The lesion showed isointense or hyperintense signals on T1-weighted images (**a**) and T2-weighted images (**b**). Contrast-enhanced T1-weighted images showed no lesion enhancement: (**c**: arterial phase; **d**: venous phase; **e**: equilibrium phase). Contrast-enhanced T1-weighted images showed postoperative remnant liver volume increased significantly in the portal vein phase and no encapsulated peritoneal effusions (**f**)

**Fig. 2:**
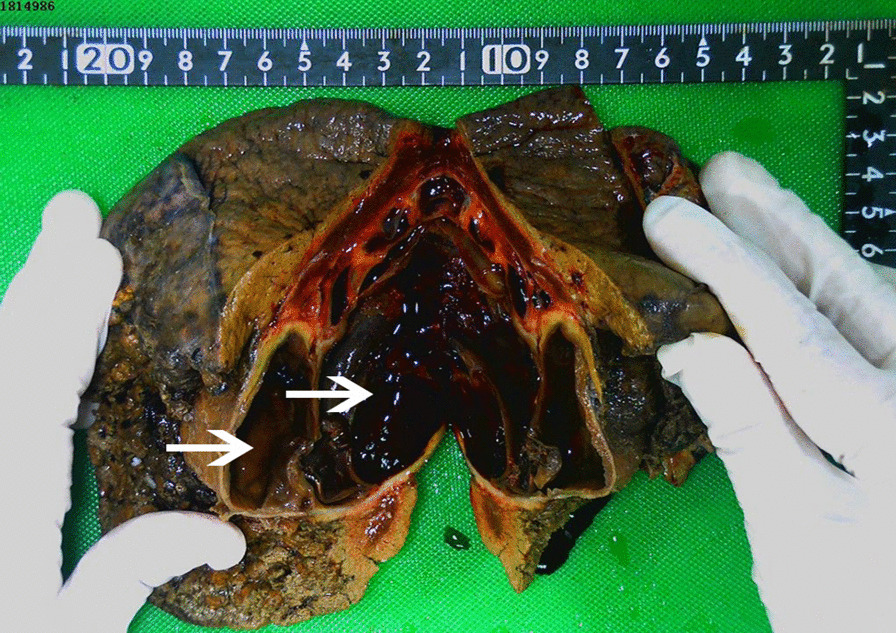
Gross photographic. Macroscopic specimen showing multiple-room and intratumoral hemorrhage within the hemangioma (white arrow)

**Fig. 3:**
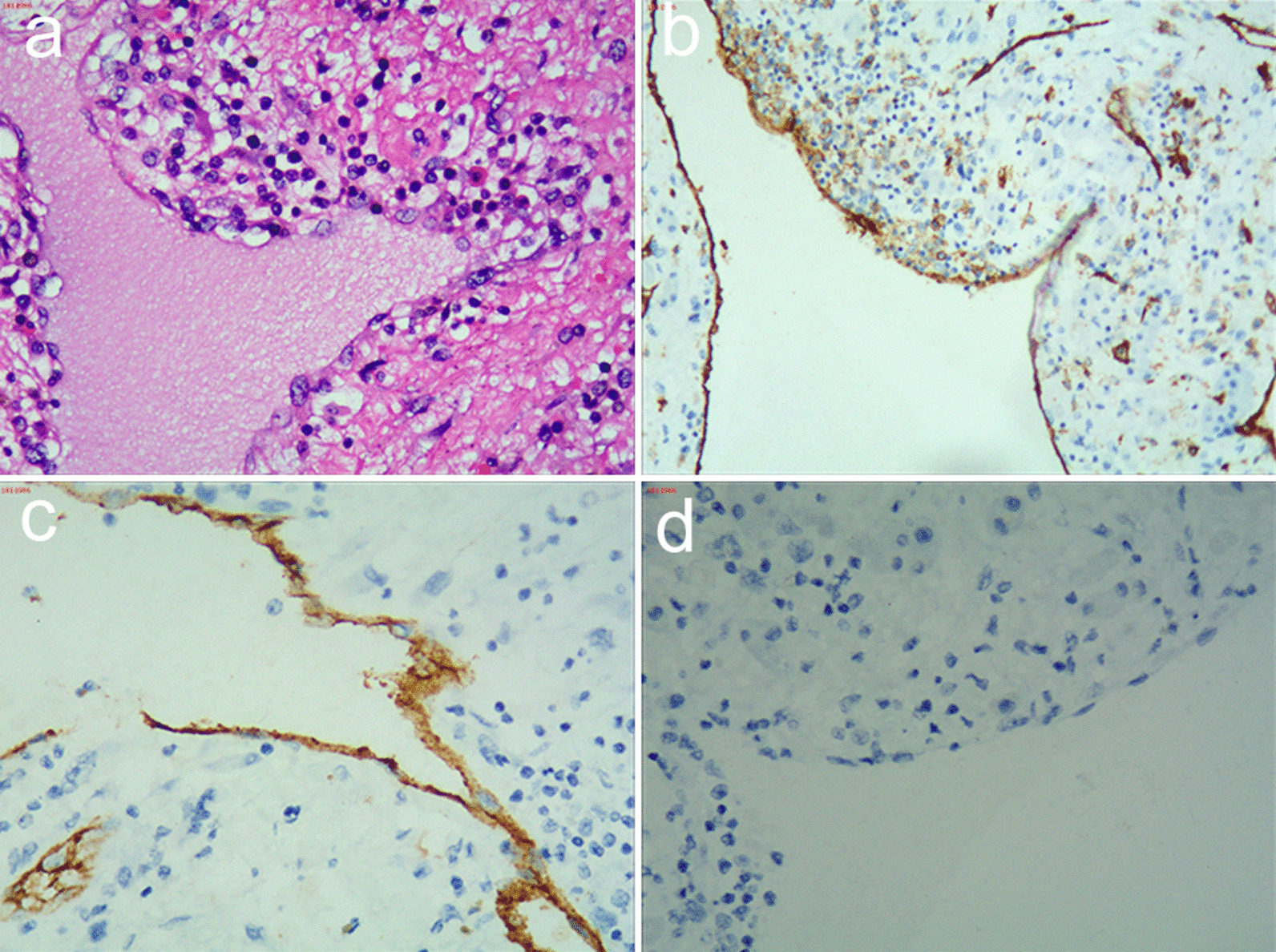
Pathological findings. (**a**) Immunohistochemically, the resected specimen was composed of dilated blood vessels lined with flat endothelial cells, contained a localized hemorrhage, and showed cystic changes [hematoxylin–eosin (HE) staining, magnification 200×]. Tumor endothelial cells stained positive for CD-31 (magnification 200×) (**b**), CD-34 (magnification 200×) (**c**), and CD-240 (magnification 200×) (**d**). Abbreviations: CD-31, cluster of differentiation-31; CD-34, cluster of differentiation-34

A 65-year-old man was referred to our hospital experiencing a persistent dull pain in the upper right abdomen lasting 5 days. Initially, he visited a local hospital, where a Color Doppler ultrasound revealed a giant cystic mass, which was more likely to undergo intracapsular hemorrhaging. It had occurred suddenly without a history of recent trauma. He had no history of viral hepatitis or a family history of cancer and recently lost weight. On physical examination, his conjunctiva was not pale, and jaundice was not observed in his sclera. His liver was not palpable below the costal margin. There was tenderness in the right liver area, a negative Murphy’s sign. Laboratory examinations showed a white blood cell count of 6.04 × 10^9^/L (normal range: 4–10 × 10^9^/L), a red blood cell count of 3.75 × 10^12^/L (normal range: 4.0–5.5 × 10^12^/L), hemoglobin level of 124 g/L (normal range: 120–180 g/L), platelet count of 279 × 10^9^/L (normal range: 100–300 × 10^9^/L), an activated partial thromboplastin time (APTT) of 31.8 s (normal range: 26.0–40.0 s), a prothrombin time (PT) of 11.0 s (normal range: 10.6–14.3 s), an alanine transaminase (ALT) level of 17 U/L (normal range: 9–50 U/L), an aspartate aminotransferase (AST) level of 17.7 U/L (normal range: 9–48 U/L), an alkaline phosphatase (ALP) level of 106 U/L (normal range: 31–115 U/L), a total bilirubin (TB) level of 5.6 μmmol/L (normal range: 2–20 μmmol/L), and an albumin (ALB) level of 37.4 g/L (normal range: 35–55 g/L). Tests revealed that HBsAg was negative, anti-HBs Ab was positive, and anti-HCV Ab was negative. Concentrations of all tumor markers were also determined and showed an alpha-fetoprotein (AFP) level of 1.65 ng/mL (normal range: 0–9 ng/mL), a carbohydrate antigen 19–9 (CA 19–9) level of 5.59 U/mL (normal range: 0–27 U/mL), and a carcinoembryonic antigen (CEA) level of 0.28 ng/mL (normal range: 0–5 ng/mL). Abdominal MRI revealed an 11.3 cm × 8.2 cm × 9.9 cm cystic mass in the right lobe of the liver, with multiple-room and an uneven signal(Fig. 1 arrow). The lesion showed isointense or hyperintense signals on T1-weighted images and on T2-weighted images (Fig. 1A-B arrows). After the contrast material agent's infusion, an unenhanced lesion was found in the cyst (Fig. 1 C-E). The preoperative diagnosis was hepatic cystic adenoma hemorrhage or hepatic cyst hemorrhage. There was no effusion in the abdominal cavity during the operation, no obvious cirrhosis, and a giant cystic mass was located in the liver's right lobe. The cystic mass's maximum diameter was about 12 cm, intact, and no bleeding was detected. The capsule of the mass was close to the bifurcation of the right portal vein. The patient underwent a right hepatic lobectomy. We observed that the resected specimen was multiple-room and found necrotic materials and hemorrhagic components (Fig. 2). The operation was successful, and the patient was administered anti-inflammation, liver protection, and pain relief. Microscopically, the tumor was composed of dilated blood vessels that were lined with flat endothelial cells, a localized hemorrhage, and cystic changes (Fig. 3a). Immunohistochemistry staining showed that endothelial cells were CD31, CD34, and D-240 positive (Fig. 3b–d). The patient recovered well and was discharged 8 days after the surgery. One month after surgery, MRI scans revealed remnant liver volume increased significantly and showed no encapsulated peritoneal effusions (Fig. 1f).

Similar articles were obtained using the keywords “hepatic hemangioma, internal hemorrhage" through searching “PubMed, National Library of Medicine, Web of Science, VIP database, China National Knowledge Infrastructure (CNKI), and Wan fang database for articles from January 1995 to January 2021. We retrieved only 8 cases reported in Chinese or English. Patient data, including author, gender, age, preoperative diagnosis, and treatment procedures, were extracted. We summarized the relevant findings in Table 1. There were seven females and one male, aged from 29 to 70 years, with a mean age of (49.3 ± 13.7) years. Lesions were located in the right lobe (n = 2), the left lobe (n = 4), left and right lobes (n = 1), and the caudate lobe (n = 1). The tumor sizes ranged from 4 cm to the whole hepatic lobe.

**Table 1:** The previously reported eight cases of spontaneous internal hemorrhage of hepatic hemangioma [7–14]

Case no	First author	Year	Age/sex	Chief complaint	Tumor location	Method of diagnostic Imaging	Preoperative diagnosis	Management
1	Wang et al. [14]	2020	59 F	Fever and cough	Right hepatic lobe	CT	Hepatic hemangioma with hemorrhage	Resection
2	Hao et al. [12]	2018	52 F	Fever	Right hepatic lobe	CT	liver abscess	TAE, then resection
3	Lucia et al. [13]	2018	70 F	Abdominal pain and fever	Left hepatic lobe	CT	liver mass	Resection
4	Kim et al. [11]	2015	54 F	Abdominal pain	Left hepatic lobe	CT	Intracapsular hemorrhage of cystic liver lesion	Resection
5	Zhang et al. [10]	2011	35 F	Abdominal mass	Left hepatic lobe	CT	Intracapsular hemorrhage of hepatic hemangioma	Resection
6	Feldman et al. [7]	2007	39 M	Abdominal pain	Left and right hepatic lobe	CT	Cystic liver lesion: liver cancer?	Resection
7	Shimoji et al. [9]	2004	56 F	Anemia and fever	Left hepatic lobe	MR	Cystic liver lesion: liver cancer?	Resection
8	Graham et al. [8]	1993	29 F	Vomiting and abdominal pain	Caudate lobe	Color Doppler ultrasound and MRI	Intracapsular hemorrhage of the liver caudate lobe: adenoma?; FNH(focal nodular hyperplasia)? or hemangioma? etc	TAE

All patients had no history of trauma. The methods of diagnostic Imaging included Computed Tomography (CT), MRI, or Ultrasound. The six patients underwent surgery directly; one patient first underwent a transcatheter arterial embolization (TAE), then agreed to liver resection, while one selected TAE due to pregnancy. All patients were successfully cured and discharged, and no patients died.

## Discussion and conclusions

Cavernous hemangioma is most common in the liver. The cause of the disease is not clear; it is believed that it may be related to abnormal vasculogenesis and angiogenesis [15]. Hepatic hemangiomas grow slowly, and the course of the disease lasts for several years. There are no clinical symptoms when the tumor is small. As the tumor size increases, it causes abdominal discomfort or pain, vomiting, fever, and belching [16]. Besides Kasabach-Merritt syndrome, most doctors often focus on abdominal hemorrhages caused by the rupture of hepatic hemangiomas. However, reports on hepatic hemangioma with internal hemorrhage are rare, causing a lack of further understanding and ignoring its serious harmfulness.

Patients with hepatic hemangioma have different clinical symptoms, and abdominal pain is the most common symptom. The majority of patients only have upper abdominal pain; however, typical hepatic hemangioma rupture symptoms include sudden, severe abdominal pain, massive abdominal bleeding or hemorrhagic shock, etc. Fever, however, is a very rare symptom. In the mentioned cases, three patients had fever symptoms, one of which was first diagnosed as a liver abscess [12], but the response to anti-inflammatory drugs was inadequate. After an ultrasound-guided liver biopsy, the tissue samples showed blood clots, showing intratumoral bleeding. Another patient diagnosed with intracapsular hemorrhage of hepatic hemangioma had fever and cough symptoms, and the anti-inflammatory effect was poor. The relevant examination excluded other infection causes, then the fever symptoms disappeared after hepatectomy. As for large hemangiomas, especially with a diameter > 10 cm, we theorize the fever's cause was necrotic changes within the hemangioma.

With the improvement of health conditions and people's health awareness, more and more patients with hemangioma of the liver have been found. Hemangioma is diagnosed with various imaging techniques such as ultrasound, CT, MRI, angiography, positron emission tomography (PET), which provide a diagnostic accuracy of > 90% [17]. MRI is more specific than CT in diagnosing subacute intratumoral hemorrhage, which shows high intensity on T1- and T2-weighted images [18]. However, it is sometimes difficult for patients without a medical history of liver hemangioma to distinguish from an intracapsular hemorrhage of hepatobiliary cystadenoma, hepatic cysts, or even liver cancer. Among the eight literatures reviewed in this paper, only 2 cases were confirmed as intratumoral hemorrhage before the operation.

Hepatic hemangiomas grow slowly, and the course of the disease lasts for several years. There are no clinical symptoms when the tumor is small. When the diameter is > 4 cm, which is categorized as a “giant hemangioma” that causes abdominal discomfort or pain as the tumor size increases [19]. The incidence of abdominal bleeding caused by ruptured hepatic hemangioma is relatively low (1–4%) [20], and the mortality is quite high (36–39%) [21]. Therefore, for patients with clinical symptoms, intense surgery demands and high-risk complications should be actively treated. The treatment methods for hepatic hemangiomas include surgical resection, TAE, liver transplantation, microwave coagulation, radiofrequency therapy, and radiological therapy [22–24]. Recently, there have been reports of drugs for hepatic hemangiomas [25]. We report a patient admitted to the hospital for right upper quadrant pain. Considering the possibility of hemorrhage in the hepatic cystadenoma, We evaluate the patient's operative tolerance and the remaining liver volume. The patient underwent right hepatic lobectomy and cholecystectomy.

Intracapsular hemorrhage of hepatic cavernous hemangiomas is very rare, which is difficult for us to distinguish from hepatic cyst masses with an intratumoral hemorrhage or even malignant liver tumors. Due to the high mortality of tumor rupture, appropriate treatments are recommended as soon as possible after diagnosis.

## Data Availability

All the data and material are from the patient’s assay and examination of Affiliated Hospital of Guangdong Medical University, which are real, credible, and available.

## Abbreviations

MRI: Magnetic resonance imaging
APTT: Activated partial thromboplastin time
PT: Prothrombin time
ALT: Alanine transaminase
AST: Aspartate aminotransferase ALP: alkaline phosphatase
TB: Total bilirubin
ALB: Albumin
AFP: Alpha fetoprotein
CA 19-9: Carbohydrate antigen 19-9
CEA: Carcinoembryonic antigen
CNKI: China National Knowledge Infrastructure
CT: Computed Tomography
TAE: Transcatheter arterial embolization
PET: Positron emission tomography
CD-31: Cluster of differentiation-31
CD-34: Cluster of differentiation-34
HE: Hematoxylin–eosin

## References

[1] Choi BY, Nguyen MH (2005). The diagnosis and management of benign hepatic tumors. J Clin Gastroenterol.

[2] Althaus S, Ashdown B, Coldwell D, Helton WS, Freeny PC (1996). Transcatheter arterial embolization of two symptomatic giant cavernous hemangiomas of the liver. Cardiovasc Intervent Radiol.

[3] Giavroglou C, Economou H, Ioannidis I (2003). Arterial embolization of giant hepatic hemangiomas. Cardiovasc Intervent Radiol.

[4] Srivastava DN, Gandhi D, Seith A, Pande GK, Sahni P (2001). Transcatheter arterial embolization in the treatment of symptomatic cavernous hemangiomas of the liver: a prospective study. Abdom Imaging.

[5] Sewell J H, Weiss K. Spontaneous rupture of hemangioma of the liver. A review of the literature and presentation of illustrative case. Arch Surg.1961;83:729–33. 10.1001/archsurg.1961.01300170085016 13911012

[6] Bel Hadj M, Marzougui M, Ben Abdeljelil N, Dhouieb R, Zakhama A, Chadly A (2020). Spontaneous rupture of a hepatic cavernous hemangioma. Am J Forensic Med Pathol.

[7] Feldman PA, Regev A (2007). Atypical giant hepatic hemangiomas with intratumoral hemorrhage. Clin Gastroenterol Hepatol.

[8] Graham E, Cohen A W, Soulen M, Faye R. Symptomatic liver hemangioma with intra-tumor hemorrhage treated by angiography and embolization during pregnancy. Obstet Gynecol.1993;81(5 ( Pt 2)):813–6. 8469482

[9] Shimoji K, Shiraishi R, Kuwatsuru A, Maehara T, Matsumoto T, Kurosaki Y (2004). Spontaneous subacute intratumoral hemorrhage of hepatic cavernous hemangioma. Abdom Imaging.

[10] Zhang X, Xu J, Gao Y, Zhao W (2012). spontaneous hemorrhagic necrosis in hepatic hemangioma: a case and literature review. Chin J Cancer Prev Treatment.

[11] Kim JM, Chung WJ, Jang BK, Hwang JS, Kim YH, Kwon JH (2015). Hemorrhagic hemangioma in the liver: a case report. World J Gastroenterol.

[12] Hao F, Yang X, Tian Y, Wang W, Ge M. Spontaneous internal hemorrhage of a giant hepatic hemangioma: a case report. Medicine (Baltimore).2017;96(47):e8702. 10.1097/MD.0000000000008702 PMC5708954 29381955

[13] Dima-Cozma LC, Bitere OR, Pantazescu AN, Gologan E, Mitu F, Rădulescu D (2018). Cavernous liver hemangioma complicated with spontaneous intratumoral hemorrhage: a case report and literature review. Rom J Morphol Embryol.

[14] Wang A, Chen H, Huang Z, Tang H, Shi H, Wen J (2020). Spontaneous internal hemorrhage of a giant hepatic hemangioma with infection: a case report and literature review. J Int Med Res.

[15] Giannitrapani L, Soresi M, La Spada E, Cervello M, D'Alessandro N, Montalto G (2006). Sex hormones and risk of liver tumor. Ann N Y Acad Sci.

[16] Liu X, Yang Z, Tan H, Zhou W, Su Y (2018). Fever of unknown origin caused by giant hepatic hemangioma. J Gastrointest Surg.

[17] Toro A, Mahfouz AE, Ardiri A, Malaguarnera M, Malaguarnera G, Loria F (2014). What is changing in indications and treatment of hepatic hemangiomas: a review. Ann Hepatol.

[18] Vilgrain V, Boulos L, Vullierme MP, Denys A, Terris B, Menu Y (2000). Imaging of atypical hemangiomas of the liver with pathologic correlation. Radiographics.

[19] Nelson RC, Chezmar JL (1990). Diagnostic approach to hepatic hemangiomas. Radiology.

[20] Gupta S, Agarwal V, Acharya AN (2012). Spontaneous rupture of a giant hepatic hemangioma-report of a case. Indian J Surg.

[21] Griffa B, Basilico V, Bellotti R, Griffa A, Senatore S, Capriata G (2005). Spontaneous rupture of giant subcapsular hemangioma of the liver with hemoperitoneum and hemorrhagic shock: a case report. Chir Ital.

[22] Trastek VF, van Heerden JA, Sheedy PN, Adson MA (1983). Cavernous hemangiomas of the liver: resect or observe?. Am J Surg.

[23] Dong W, Qiu B, Xu H, He L (2019). Invasive management of symptomatic hepatic hemangioma. Eur J Gastroenterol Hepatol.

[24] Prodromidou A, Machairas N, Garoufalia Z, Kostakis ID, Tsaparas P, Paspala A (2019). Liver transplantation for giant hepatic hemangioma: a systematic review. Transplant Proc.

[25] Yamashita S, Okita K, Harada K, Hirano A, Kimura T, Kato A (2013). Giant cavernous hepatic hemangioma shrunk by use of sorafenib. Clin J Gastroenterol.

